# Fungal keratitis in Iran: Risk factors, clinical features, and mycological profile

**DOI:** 10.3389/fcimb.2023.1094182

**Published:** 2023-01-30

**Authors:** Mohammad Soleimani, Alireza Izadi, Sadegh Khodavaisy, Claudy Oliveira dos Santos, Marlou C. Tehupeiory-Kooreman, Roshanak Daie Ghazvini, Seyed Jamal Hashemi, Seyed Amin Ayatollahi Mousavi, Farzad Aala, Mahsa Abdorahimi, Mehdi Aminizadeh, Zohre Abedinifar, Shahram Mahmoudi, Afsaneh Mohamadi, Sara Rezaie, Paul E. Verweij

**Affiliations:** ^1^ Department of Ocular Trauma and Emergency, Farabi Eye Hospital, Tehran University of Medical Sciences, Tehran, Iran; ^2^ Department of Medical Parasitology and Mycology, School of Medicine, Kerman University of Medical Sciences, Kerman, Iran; ^3^ Medical Mycology and Bacteriology Research Center, Kerman University of Medical Sciences, Kerman, Iran; ^4^ Department of Medical Parasitology and Mycology, School of Public Health, Tehran University of Medical Sciences, Tehran, Iran; ^5^ Centre for Expertise in Mycology, Department of Medical Microbiology, Radboud University Medical Center, Nijmegen, Netherlands; ^6^ Laboratory of Clinical Microbiology and Infectious Diseases, Isala Hospital, Zwolle, Netherlands; ^7^ Department of Parasitology and Mycology, Faculty of Medicine, Kurdistan University of Medical Sciences, Sanandaj, Iran; ^8^ Department of Parasitology and Mycology, School of Medicine, Iran University of Medical Sciences, Tehran, Iran; ^9^ Department of Chemistry and Biology, Ryerson University, Toronto, Canada; ^10^ Department of Microbiology, Shahr-e-Qods Branch, Islamic Azad University, Tehran, Iran

**Keywords:** fungal keratitis, *Fusarium*, *Aspergillus*, antifungal susceptibility, risk factors, Iran

## Abstract

**Introduction:**

This study was intended to investigate the clinical features and predisposing factors of fungal keratitis (FK), as well as molecular identification and antifungal susceptibility of causative agents in Tehran, Iran.

**Methods:**

This cross-sectional study was carried out from April 2019 to May 2021. All fungi isolates were identified using conventional methods and were confirmed by DNA-PCR-based molecular assays. Matrix-assisted laser desorption/ionization-time of flight (MALDI-TOF) was used to identify yeast species. Minimum inhibitory concentrations (MIC) of eight antifungal agents were assessed according to the European Committee on Antimicrobial Susceptibility Testing (EUCAST) microbroth dilution reference method.

**Results:**

Fungal etiology was confirmed in 86 (7.23%) of 1189 corneal ulcers. A significant predisposing factor for FK was ocular trauma caused by plant materials. Therapeutic penetrating keratoplasty (PKP) was required in 60.4% of cases. The predominant fungal species isolated was *Fusarium* spp. (39.5%) followed by *Aspergillus* spp. (32.5%) and *Candida* spp. (16.2%).

**Discussion:**

The MIC results indicate that amphotericin B may be appropriate for treating FK caused by *Fusarium* species. FK caused by *Candida* spp. can be treated with flucytosine, voriconazole, posaconazole, miconazole, and caspofungin. In developing countries such as Iran, corneal infection due to filamentous fungi is a common cause of corneal damage. In this region, fungal keratitis is observed primarily within the context of agricultural activity and subsequent ocular trauma. Fungal keratitis can be managed better with understanding the ”local“ etiologies and antifungal susceptibility patterns.

## Introduction

Fungal keratitis (FK) is a serious eye condition caused by a variety of fungi (Ghosh et al., 2016). It can be caused by a variety of molds and yeasts, and the etiology and epidemiology of the condition may depend on geography and climatic conditions (Kredics et al., 2015; Ho et al., 2016). There are approximately 70 genera of fungi implicated in FK, but *Fusarium* and *Aspergillus* species account for 70% of the cases (Bharathi et al., 2003; Ahmadikia et al., 2021). While FK is relatively uncommon among developed countries, it is a major problem for ophthalmologist in developing countries that have tropical or subtropical climates (Kredics et al., 2015). The incidence of FK varies around the world. For instance, in Paraguay, fungi cause 50.06% of all microbial keratitis, while in Ireland, they cause 1.1% (Ahmadikia et al., 2021). FK usually results from trauma during cultivation or harvesting, so soil and plant-related workers are at a great risk (Kredics et al., 2015; Shah et al., 2017). Additionally, wide-spread use of broad-spectrum antibiotics and steroids, corneal surface diseases, frequent contact lens use or prior corneal surgery may all contribute to FK (Ng et al., 2013). The treatment of FK depends on the etiological agents, the extent of involvement, risk factors, and response to treatment of the causative agents (Puig et al., 2020). Delayed diagnosis, the limited number of available antifungal drugs, and drug resistance of the causative agents are contributing factors to treatment failure and blindness in some infected individuals (Henry et al., 2012; Al-Hatmi et al., 2014). An effective strategy for corneal ulcer management requires a thorough understanding of the local etiology of a given region. A few studies have reported the epidemiology of corneal ulceration, causative micro-organisms, and effective treatment in Iran (Mahmoudi et al., 2018; Ahmadikia et al., 2021). To provide comprehensive and valid background information and to facilitate future treatment of this infection, we conducted the present study to determine the proportion of FK among infective keratitis, risk factors, clinical outcomes, causative organisms and their drug sensitivity in the main referral center for eye disease in Tehran, Iran.

## Materials and methods

### Patients

A cross-sectional study was carried out at Farabi eye hospital, a tertiary care referral centre in Tehran, Iran from April 2019 to May 2021. We evaluated 1189 patients who had clinical suspicion of corneal ulcerations. A corneal ulcer was defined as loss of corneal epithelium with a stromal infiltrate and suppuration that are associated with signs of inflammation, with or without hypopyon. In addition to viral infections and healing ulcers, Mooren’s ulcers, interstitial keratitis, sterile neurotropic ulcers, and ulcers associated with autoimmune conditions were excluded from the study (Bharathi et al., 2003). A standardized form was filled out for each patient, documenting the patient’s sociodemographic information, the duration of symptoms, predisposing factors, any history of corneal trauma and trauma-causing agents, any associated ocular conditions, other systemic diseases, treatments received prior to presentation, and the patient’s visual acuity at the time of visit. To participate in the study, all patients provided a signed written informed consent. This study was approved by ethics committee of Tehran University of Medical Sciences (IR.TUMS.MEDICINE.REC.1400.304).

### Clinical examinations

The patients received a slit lamp biomicroscopic examination by experienced ophthalmologists. Clinical features such as the size of the epithelial defect (measured in millimetre), the size and depth of the stromal infiltrate, the presence or absence of a hypopyon (measured in millimetre), pre-existing viral keratitis and chronic corneal disease were noted. Also, we noted the use of contact lenses and topical corticosteroids and antibiotics, as well as other systemic combinations. After a detailed ocular examination, corneal scraping was performed by an ophthalmologist under aseptic conditions from each ulcer using a sterile blade (no. 15), following the instillation of local anesthetic (4% lignocaine (lidocaine)). Under magnification of a slit lamp, the procedure was performed. The material obtained by scraping spread thinly onto two labeled glass slides for 10% KOH wet mount and Gram’s staining. Furthermore, the corneal material was inoculated directly onto 5% sheep’s blood agar (BA) (Merck, Germany) and Sabouraud dextrose agar (SDA) (Merck, Germany). BA plates incubated at 37°C and SDA plates were incubated at 27°C aerobically. The cultures examined daily, and discarded at 3 weeks if no growth was seen.

### Laboratory investigation

The diagnosis of FK was made if fungi were seen in direct microscopic examination and grew in one of the culture media. The isolated fungi were identified at the Mycology Reference Laboratory of Tehran University of Medical Sciences and Center of Expertise in Mycology, Radboud University Medical Center, Nijmegen, Netherlands. In addition to the macroscopic and microscopic appearances, a molecular method was used to identify filamentous and yeast fungi. Standard protocols were followed for all laboratory procedures. We stored all isolates in tryptic soy broth (TSB; Liofilchem, Italy) supplemented with glycerol at -20°C for further evaluation.

### Isolates identification

The genomic DNA of all strains was extracted from fresh colonies using a DNA isolation kit (Gene All DNA extraction kit; Gene All, Germany) according to the manufacturer’s instructions. The extraction products were electrophoresed on 1% agarose gel and stored at -20°C. TEF-1α, β-tubulin and ITS1-5.8SrDNA-ITS2 partial genes were amplified for the identification of *Fusarium* spp., *Aspergillus* spp. and other molds, respectively. The PCR products were sequenced and analyzed using the non-redundant nucleic databases BLAST (http://www.ncbi.nim.nih.gov/BLAST/). The identification of yeast fungi was accomplished using Bruker MALDI-TOF MS apparatus and software at the Center of Expertise in Mycology Radboud University Medical Center/CWZ, Nijmegen, Netherlands.

### *In vitro* antifungal susceptibilities testing

For assessing antifungal susceptibility, broth microdilutions performed in sterile, flat-bottomed 96-well microplates. The test medium was RPMI-1640 (Gibco). Susceptibility of fungal isolates to fluconazole (range 64–0.06 µg/ml) (Pfizer, Groton, CT, USA), 5-flucytosine (range 64–0.06 µg/ml) (Sigma, St. Louis, MO, USA), voriconazole (range 16–0.03 µg/ml) (Pfizer, Groton, CT, USA), posaconazole (range 16–0.03 µg/ml) (Pfizer, Groton, CT, USA), miconazole (range 16–0.03 µg/ml) (Pfizer, Groton, CT, USA), natamycin (range 16–0.03 µg/ml) (Sigma, St. Louis, MO, USA), amphotericin B (range 16–0.03 µg/ml) (Sigma, St. Louis, MO, USA), and caspofungin (range 8–0.008 µg/ml) (Sigma, St. Louis, MO, USA) was evaluated. The MICs were determined using EUCAST microdilution reference method (EUCAST –EDef 9.3 for filamentous fungi and EUCAST –EDef 7.3 for yeast) (Arendrup et al., 2015b; Arendrup et al., 2015a). The fungi suspensions were obtained from fresh cultures. Cell density was adjusted to 1 x 10^5^ cfu/mL to 2.5 x 10^5^ cfu/mL for filamentous fungi and 0.5 x 10^5^ to 2.5 x 10^5^ CFU/mL for yeast isolates, by spectrophotometer. *Candida krusei* (ATCC 6258), *Candida parapsilosis* (ATCC 22019), *Aspergillus fumigatus* (ATCC 204305) and *Aspergillus flavus* (ATCC 204304) was used as a quality control strain. A 100µl aliquot of each antifungal was dispensed into wells, and then 100 µl of fungal suspension was added to each well. The microplates were incubated for 24 and 48 hours at 37°C, and MICs were determined after that time. All tests were performed in duplicate. In contrast to the control, the lowest inhibitory concentration that exhibited a >90% reduction in turbidity for the polyenes was defined as the MIC. A turbidity reduction of >50% was considered the end point for azoles. Caspofungin cutoffs were determined based on the minimum effective concentration (MEC), or the lowest concentration that induces aberrant growth (Arendrup et al., 2015b; Arendrup et al., 2015a).

### Statistical analyses

The statistical analysis was performed by Statistical Package for Social Sciences (SPSS) V. 22.0 for Windows (SPSS Inc., Chicago, IL, USA). The MIC_50_/MIC_90_/GM and frequency of studied isolates were determined. Data analysis was performed using the T-test. *P values ≤*0.05 were considered statistically significant.

## Results

Of the 1189 patients with infective corneal ulcers, 86 (7.23%) were diagnosed as FK. Of the 86 patients, 57 (66.2%) were male, and 29 (33.8%) were female (P< 0.05). The male-to-female ratio was1.9:1 and the age of patients ranged from 22 to 103 years (mean; 52.9). The most frequent age group was 25-50 (41; 47.6%). The majority of patients (32/86; 37.2%) were farm workers. Trauma was noted as the predominant predisposing factor in 42 (48.8%) patients. Plant debris and stalks were the most common traumatic agents (21/86; 24.4%). Seasonal variation showed that autumn had a higher proportion of cases compared to other seasons. (34/86; 39.5%) (P<0.05). **Table 1** presents the demographic characteristics, predisposing factors, and seasonal distribution.

**Table 1:** Demographic characteristics, predisposing factors and traumatic agents in patients with fungal keratitis.

Particulars		Frequency	Percentage
Gender	Male	57	66.2%
	Female	29	33.8%
Age groups (Year)	<25	7	8.1%
	25-50	41	47.6%
	51-75	32	37.2%
	>75	6	6.9%
Occupation	Farm worker	32	37.2%
	Laborer	18	20.9%
	Household	23	26.7%
	Unemployed	4	4.6%
	Other	9	10.4%
Predisposing factor	Trauma	42	48.8%
	Ocular surgery	10	11.6%
	Diabetes mellitus	6	6.9%
	Use of contact lens	5	5.8%
	History of infective keratitis	5	5.8
	Local corneal disease	4	4.6%
	Keratoplasty	4	4.6%
	Use of topical steroids	3	3.5%
	Cancer	1	1.1%
	Unknown	6	6.9%
Traumatic agents	Plant debris	21	24.4%
	Soil/Sand/Stone	10	11.6%
	Metal particle	6	6.9%
	Unknown	5	5.8%
Seasonal distribution	Autumn	34	39.5%
	Summer	27	31.3%
	Spring	13	15.1%
	Winter	12	13.9%

### Clinical features

All the 86 cases of FK were unilateral and 47 (55%) of them were in the right eye. The diameter of corneal ulcers ranged from 1 to 11 mm (mean; 4.05 mm). The corneal ulcer in 75 (87.2%) patients was 2-6 mm. Visual acuity at first consultation in a majority of cases (35/86; 40.6%) was light conception to hand motion. Slit lamp evaluation revealed hypopyon in 56 (65.11%), feathery pattern in 59 (68.6%) and satellite lesion in 17 (19.7%) patients. Blurred vision, redness, watering, pain and photophobia noted for all patients (**Table 2**).

**Table 2:** Clinical feature of patients with fungal keratitis.

Clinical feature		Number	Percentage
Site of FK	Right eye	47	55
	Left eye	39	45
Diameter of corneal ulcers	<2mm	3	3.4
	2-6mm	75	87.2
	>6mm	8	9.3
Visual acuity	LC-HM	35	40.6
	FC-20/200	26	30.2
	20/200-20/20	19	22.1
Hypopyon		56	65.1
Feathery pattern		59	68.6
Satellite lesion		17	19.7

FK, Fungal keratitis; LC, Light conception; HM, Hand motion; FC, Finger count.

### Spectrum of pathogenic fungi

Considering the conventional and molecular approaches *Fusarium* spp. were the most frequent isolates (34/86; 39.5%), followed by *Aspergillus* spp. (28/86; 32.5%) and *Candida* spp. (14/86; 16.2%). The identified *Fusarium* species belonged to four species complexes (SC); the most common SC was *F. solani* (FSSC; n=27), followed by *F. fujikuroi* (FFSC; n=4), *F. dimerum* (FDSC; n=2) and *F.sambucinum* (FSAMSC; n=1). *Aspergillus* isolates analyzed by β-tubulin sequencing belong to 4 sections; section Flavi (n=21), section Nigri (n=5), section Terrei (*A. terreus*; n=1) and section Circumdati (*A. ochraceus*; n=1). The most common *Candida* spp. were *C. albicans* (10/86; 11.6%) followed by *C. parapsilosis* (4/86; 4.6%) (**Table 3**).

**Table 3:** Fungal pathogens isolated from 86 cases of fungal keratitis.

Species	No.	Percentage
*Fusarium* species complex	34	39.5%
FSSC		
*F. solani*	26	30.2%
*F. falciforme*	1	1.1%
FFSC		
*F. proliferatum*	2	2.3%
*F. acutatum*	1	1.1%
*F. thapsinum*	1	1.1%
FDSC		
*F. delphinoides*	2	2.3%
FSAMSC		
*F. brachygibbosum*	1	1.1%
*Aspergillus* species	28	32.5%
*Section flavi (A. flavus)*	21	24.4%
*Section nigri (A. niger)*	5	5.8%
*Section terrei (A. terreus)*	1	1.1%
*Section circumdati (A. ochraceus)*	1	1.1%
*Candida* species	14	16.2%
*C. albicans*	10	11.6%
*C. parapsilosis*	4	4.6%
*Scedosporium apiospermum*	4	4.6%
*Curvularia spicifera*	1	1.1%
*Bipolaris panici-miliacei*	1	1.1%
*Alternaria species*	1	1.1%
*Alternaria alternata*	1	1.1%
*Geotrichum capitatum*	1	1.1%
*Colletotrichum gloeosporioides*	1	1.1%
Total	86	100%

FSSC, F. solani species complex; FFSC, F. fujikuroi species complex; FDSC, F. dimerum species complex; FSAMSC, F.sambucinum species complex.

### Treatment and outcome

All 86 patients were hospitalized and received antifungal treatment. Voriconazole 1% was applied topically to 35 (38.5%) patients. It was the most commonly used antifungal, either alone or in combination with other antifungals. Surgical treatment were performed in 65 patients (75.6%) (**Table 4**). Of the 86 patients treated with antifungal and surgery, 11 (3.5%) developed recurrent fungal infections. *Fusarium* species (n=6), including *F. solani* (n=5) and *F. brachygibbosum* (n=1), were the most common pathogens detected here, followed by *A. flavus* (n=3), *C. spicifera* (n=1) and *C. parapsilosis* (n=1). In two patients, *F. solani* was isolated three times.

**Table 4:** Treatment of fungal keratitis in studied patients.

Treatment	Frequency
Antifungal treatment	
Topical VCZ 1% + Topical AMB 0.1%	61(70.9%)
Oral ICZ (200mg/day)+ Topical VCZ 1% + Topical AMB 0.1%	55(63.9%)
Oral ICZ (200mg/day)+ Topical VCZ 1%	47(54.6%)
Topical VCZ 1%	35(40.7%)
Topical AMB 0.1% + topical FLZ	8(9.3%)
Surgical treatment	65(75.6%)
Therapeutic PK	51(59.3%)
Corneal glue	10(11.6%)
Patch graft	2(2.3%)
AMT	2(2.3%)

VCZ, Voriconazole; ICZ, Itraconazole; AMB, Amphotricin B; FLZ, Fluconazole; PK, penetrating keratoplasty; AMT, amniotic membrane transplantation.

### Antifungal susceptibility pattern

All *Fusarium* isolates were inhibited by ≤ 4 μg/ml amphotericin B, while fewer isolates were inhibited by similar levels of voriconazole (20.5%), posaconazole (17.6%), caspofungin (11.7%), and natamycin (44.1%). The highest MIC in *Fusarium* isolates recorded was 64 μg/ml for fluconazole and 5- flucytosine. At ≤ 4 μg/ml concentrations, *Aspergillus* isolates were inhibited by Amphotericin B (96.4%), 5-flucytosine (32.1%), voriconazole (100%), posaconazole (100%), miconazole (82.1%), caspofungin (100%) and natamycin (35.7%). All *Aspergillus* isolates showed a MIC of 64 μg/ml to fluconazole. All *Candida* species were inhibited by amphotericin B, 5- flucytosine, voriconazole, posaconazole, miconazole and caspofungin when applied at a concentration of ≤ 1 μg/ml, while a lesser number of isolates were inhibited by fluconazole at similar concentrations (85.7%). Based on the break point suggested by EUCAST, one *C. albicans* and one *C. parapsilosis* were resistance to fluconazole (MIC, 8 μg/ml). Moreover, one *C. albicans* and one *C. parapsilosis* were dose dependent to voriconazole (MIC, 0.25 μg/ml). Approximately all of the *Scedosporium apiospermum* and dematiaceous fungi were inhibited at ≤ 4 μg/ml concentrations of amphotericin B, voriconazole, posaconazole, miconazole, caspofungin and natamycin, while 5-flucytosine and fluconazole inhibited these fungi at 64 μg/ml. Currently, only a limited number of species-specific break-points (BP) and epidemiological cutoff values (ECV) have been established by the EUCAST for testing the susceptibilities of fungi and antifungal agents. As a result of insufficient data, ECVs and BPs were not considered for all species evaluated, but their pooled MIC distributions were included in **Table 5**.

**Table 5:** Antifungal Susceptibility Pattern of assayed isolates.

Isolates (No.)	Antifungal agent	MIC range	MIC_50_	MIC_90_	GM	MIC range										
						<0.06	0.125	0.25	0.5	1	2	4	8	16	32	64
*Fusarium* Spp. (34)	AMB	**025-4**	**1**	**4**	**1.35**			**1**	**3**	17	**8**	**5**				
	5-FC	**65**	**64**	**64**	**64**											34
	FLZ	**64**	**64**	**64**	**64**											34
	VRZ	**1-8**	**8**	**8**	**6.34**					**1**	**2**	**4**	27			
	PSZ	**1-8**	**8**	**8**	**6.96**					**3**	**1**	**2**	28			
	MCZ	**16**	**16**	**16**	**16**									34		
	CSP	**4-16**	**16**	**16**	**14.25**							**4**	**3**	27		
	NTM	**2-8**	**8**	**8**	**5.65**						**3**	**12**	19			
*Aspergillus* Spp. (28)	AMB	**0.125-16**	**2**	**2**	**1.15**		**1**	**3**	**1**	**6**	10	**6**		**1**		
	5-FC	**2-64**	**64**	**64**	**29.23**						**4**	**5**	**1**	**2**		16
	FLZ	**64**	**64**	**64**	**64**											28
	VRZ	**0.125-1**	**0.5**	**1**	**0.59**		**1**		19	**8**						
	PSZ	**0.016-0.125**	**0.063**	**0.125**	**0.07**	15	**8**	**5**								
	MCZ	**1-8**	**4**	**8**	**4.24**					**1**	**2**	20	**5**			
	CSP	**0.125-0.5**	**0.25**	**0.25**	**0.22**		**5**	21	**2**							
	NTM	**2-16**	**16**	**16**	**10.62**						**3**	**7**		18		
*Candida* Spp. (n=14)	AMB	**0.125-1**	**0.5**	**0.5**	**0.43**		**1**	**2**	10	**1**						
	5-FC	**0.063-0.25**	**0.125**	**0.22**	**0.112**	**4**	7	**3**								
	FLZ	**0.125-8**	**0.125**	**0.75**	**0.304**		8	**2**	**2**				**2**			
	VRZ	**0.008-1**	**0.008**	**0.17**	**0.015**	12		**2**								
	PSZ	**0.008-2**	**0.008**	**0.04**	**0.013**	13		**1**								
	MCZ	**0.016-0.125**	**0.016**	**0.125**	**0.033**	10	**4**									
	CSP	**0.125-1**	**0.25**	**1**	**0.371**		**1**	8	**1**	**4**						
	NTM	**2-8**	**4**	**4**	**4**						**1**	12	**1**			
*Scedosporium apiospermum* (n=4)	AMB	**4**	**4**	**4**	**4**							4				
	5-FC	**64**	**64**	**64**	**64**											4
	FLZ	**64**	**64**	**64**	**64**											4
	VRZ	**0.25-0.5**	**0.5**	**0.5**	**0.42**			**1**	3							
	PSZ	**0.5-1**	**1**	**1**	**0.84**				**1**	3						
	MCZ	**0.5**	**0.5**	**0.5**	**0.5**				4							
	CSP	**0.25-2**	**0.5**	**2**	**0.594**			**2**	**2**							
	NTM	**2-4**	**2**	**4**	**2.378**						3	**1**				
Dematiaceous (n=4)	AMB	**0.125-4**	**0.125**	**4**	**0.396**		3					**1**				
	5-FC	**64**	**64**	**64**	**64**											4
	FLZ	**64**	**64**	**64**	**64**											4
	VRZ	**2**	**2**	**2**	**2**						4					
	PSZ	**0.125-0.25**	**0.125**	**0.225**	**0.157**		3	**1**								
	MCZ	**4-8**	**4**	**8**	**5.039**							3	**1**			
	CSP	**0.125-0.5**	**0.25**	**0.45**	**0.25**		**1**	**2**	**1**							
	NTM	**0.008-0.016**	**0.008**	**0.014**	**0.01**	4										
*Colletotrichum gloeosporioides* (n=1)	AMB	**0.5**	**-**	**-**	–				**1**							
	5-FC	**32**	**-**	**-**	–										**1**	
	FLZ	**64**	**-**	**-**	–											**1**
	VRZ	**1**	**-**	**-**	–					**1**						
	PSZ	**1**	**-**	**-**	–					**1**						
	MCZ	**2**	**-**	**-**	–						**1**					
	CSP	**1**	**-**	**-**	–					**1**						
	NTM	**2**	**-**	**-**	–						**1**					
*Geotrichum capitatum* (n=1)	AMB	**1**	**-**	**-**	–					**1**						
	5-FC	**0.25**	**-**	**-**	–			**1**								
	FLZ	**16**	**-**	**-**	–									**1**		
	VRZ	**1**	**-**	**-**	–					**1**						
	PSZ	**2**	**-**	**-**	–						**1**					
	MCZ	**4**	**-**	**-**	–							**1**				
	CSP	**16**	**-**	**-**	–									**1**		
	NTM	**8**	**-**	**-**	–								**1**			

AMB, Amphotericin B; 5-FC, 5- Flucytosine; FLZ, Fluconazole; VRZ, Voriconazole; PSZ, Posaconazole; MCZ, Miconazole; CSP, Caspofungin; NTM, Natamycin; Numbers in bold are modal value.

## Discussion

Keratitis is the second leading cause of blindness worldwide and the most common cause of vision disorders and complications (Bharathi et al., 2003; Gupta et al., 2014). FK is a serious ophthalmological condition in all parts of the world. In the case of delayed diagnoses and improper treatment, FK can cause severe corneal damage and even blindness. Based on various published reports, FK accounts for 6 to 50% of ulcerative keratitis cases (Thomas, 2003; Srinivasan, 2004; Shokouhi et al., 2006). Although the disease is found worldwide, it is most prevalent in tropical and subtropical regions, especially among farmers (Shukla et al., 2008). The results of this study indicated that 86 (7.23%) of 1189 patients with suspected infectious keratitis were diagnosed with FK. According to the studies conducted in India, Egypt and Saudi Arabia FK accounted for 34.4%, 43.3% and 3.8% of microbial keratitis, respectively (Saha and Das, 2006; Alkatan et al., 2012; Khater et al., 2014). The incidence of FK can vary depending on the region of a country; for example, it was 32%, 38.9%, 32%, and 39.8% in North, East, West, and South India, respectively (Chowdhary and Singh, 2005). Studies conducted in Iran have found that the incidence of FK varies from region to region. In Sari (northern Iran), Shokouhi et al. (2006) reported an incidence of FK of 31.8% (Shokouhi et al., 2006), whereas another study from Tehran (2012) reported an incidence of 5.5% (Ebadollahi-Natanzi et al., 2016). Similarly, in Rasht in north of Iran (2017), the incidence of FK was reported to be 16% (Tighnavard et al., 2017). Houang et al. (2001) explored the relationship between FK and climate, they concluded that although higher levels of fungal keratitis could be expected in regions with more rainfall and temperatures, this was not always the case and depended on other factors (Houang et al., 2001). There are few cases of FK without associated predisposing factors (Mahmoudi et al., 2018). Some of the predisposing factors for FK include trauma, immunodeficiency, ocular surface disorders, ocular surgery, treatment with topical steroids, and long-term use of soft contact lenses (Cheikhrouhou et al., 2014). According to different studies, corneal trauma (primarily vegetative matter) is the predominant cause of FK in 40-60% of cases (Mahmoudi et al., 2018). The results of our study revealed that corneal trauma was the most important factor predisposing patients to FK (42/86, 48.8%) followed by eye surgery (10/86, 11.6%). Additionally, we found that plant organs were the most important traumatic factor among our patients. There have been similar findings reported in Melbourne, the southern United States, Singapore, India, and Bangladesh, as well as in previous studies in Iran (Shokouhi et al., 2006; Cheikhrouhou et al., 2014; Ebadollahi-Natanzi et al., 2016; Tighnavard et al., 2017; Lee et al., 2018; Mahmoudi et al., 2018). In contrast, eye trauma was reported as the second risk factor in the northern United States (Keay et al., 2011). Additionally, a study in Philadelphia noted that chronic eye disease, the habit of wearing contact lenses, and the use of topical corticosteroids are three common underlying factors (Tanure et al., 2000). A significant increase in FK occurs in men from rural areas following corneal damage (Bharathi et al., 2003). In this study, the male to female ratio was 1.9: 1 (male: 57/86, 66.2%). Furthermore, most FK patients (41/86, 47.6%) were aged 25 to 50 and were farmers. Different studies report higher incidences of FK in the 35-59 age group and among farmers, as well as a higher ratio of males to females (Saha and Das, 2006; Chander et al., 2008; Satpathy et al., 2019). Since this age group is more active outdoors and is in contact with soil and nature, they are more exposed to fungal agents (Cheikhrouhou et al., 2014). Similar findings have been observed in other studies conducted in Iran (Shokouhi et al., 2006; Ebadollahi-Natanzi et al., 2016; Tabatabaei et al., 2018). There is a higher prevalence of FK in the autumn and during the year when more agricultural activities occur (Bharathi et al., 2003). The highest proportion of cases in each season was noted in autumn (34/86, 39.5%) in our study. Several studies have demonstrated a significant increase in the number of reported cases of FK during harvest season and the monsoon winds (Gopinathan et al., 2002; Bharathi et al., 2003; Mahmoudi et al., 2018). Despite its widespread incidence, FK has no consistent clinical picture (Fong et al., 2004; Dahlgren et al., 2007). Clinical signs that can assist with diagnosing FK include severe hypopyon, lesions with feathery margins, corneal perforation, and satellite lesions (Mahmoudi et al., 2018; Satpathy et al., 2019). We found hypopyon, feathery margins, and satellite lesions on the slit-lamp examination in 56 (65.11%), 59 (68.6%) and 17 (19.7%) of our patients, respectively. Furthermore, the corneal ulcer diameter ranged from 1 to 11 mm (mean: 4.05 mm). As reported by Bharati et al., feathery margins were found in 786 (71.78%), satellite lesions in 110 (10.05%), and hypopyon in 609 (55.62%) cases of FK (Bharathi et al., 2003). Rosa et al. observed feathery margins in 62% of their patients as well as a rough texture in 47%, and satellite lesions in 41% (Rosa et al., 1994). In contrast, Shokohi et al. observed only one feathery pattern and did not observe hypopyon and satellite lesions among their FK patients (Shokouhi et al., 2006). As described above, these findings are not pathognomonic, and can lead to misdiagnosis and treatment with antiviral, antibacterial or corticosteroid drugs (Satpathy et al., 2019).

In most cases, FK is treated by topical administration of anti-fungal medications, but there are no gold standards (Iselin et al., 2017). Because of the limited availability of antifungal drugs and the emergence of resistant species, the treatment of FK has become a major challenge. Without proper treatment, it can lead to vision loss in cases of deep lesions (Sharma et al., 2011). Natamycin was previously an effective drug for treating FK, however, poor penetration of this drug into the corneal stroma has led to reports of treatment failure for this drug. As an alternative, topical amphotericin B 0.3% to 0.5% and voriconazole 1% are recommended (Sharma et al., 2011; Sharma et al., 2016). In the present study, 62.8% of patients received a combination of voriconazole, itraconazole, and amphotericin B. In 32 out of 86 patients, drug treatment did not improve the condition, so penetrating keratoplasty was employed. Among the patients who received antifungal and surgical treatment, 11 (3%) developed recurrent FK. A combination of systemic voriconazole and topical natamycin was found to be one of the most commonly recommended antifungal treatment regimens by Iselin et al. (Iselin et al., 2017). A report by Ghosh et al. noted that 95% of patients with FK were treated with either topical preparations of natamycin alone or in combination with other topical antifungals, including itraconazole, fluconazole, and voriconazole (Ghosh et al., 2016) . Tanure et al. used penetrating keratoplasty on six patients with acute fungal keratitis while they were being treated with combination of natamycin and amphotericin B (Tanure et al., 2000). According to previous studies, keratoplasty is required in 26 to 35% of cases of acute FK (Sanders, 1970). An accurate diagnosis of the causative agents for fungal infections is crucial for proper treatment. Improvements in diagnostic methods are essential for this (Fong et al., 2004). FK can be caused by yeasts or molds (Bharathi et al., 2003). FK due to molds is more common in rural areas among agricultural workers and in urban areas among construction workers. Yeast keratitis, on the other hand, usually affects patients with chronic systemic diseases, viral eye infections, and contact lens wearers (Satpathy et al., 2019). We found 72 filamentous fungi (83.7%) and 14 yeast cases (16.3%) in our study. As reported in most studies, *Fusarium* spp., *Aspergillus* spp., and *Candida* spp. are the most common pathogens associated with FK (Ahmadikia et al., 2021). In our study, *Fusarium* spp. were the most frequent isolates (34/86; 39.5%), followed by *Aspergillus* spp. (28/86; 32.5%) and *Candida* spp. (14/86; 16.2%). The most common cause of FK in India, Nepal, Sri Lanka, and Bangladesh has consistently been identified as *Aspergillus* spp. (Mahmoudi et al., 2018). *Fusarium* spp. were the most common causative agents in Africa and China (Xie et al., 2006; Thomas and Kaliamurthy, 2013). In European countries, FK is rare and *Candida* spp. are the most common isolates (Ahmadikia et al., 2021). There are other Iranian studies that report *Aspergillus* spp. and *Fusarium* spp. being the most common fungal pathogens in FK (Shokouhi et al., 2006; Ebadollahi-Natanzi et al., 2016; Tighnavard et al., 2017). There is a lack of information about the abundance of fungal species known to cause fungal keratitis in Iran due to the limited number of molecular identifications carried out in previous studies. In this study, PCR-based sequencing revealed that *F. solani* (26/86; 30.2%) and *A. flavus* (21/86; 24.4%) were the most frequently identified species. Additionally, MALDI-TOF results showed that the most frequent *Candida* species was *C. albicans* (10/86; 13%). Although *in vitro* antifungal susceptibility testing cannot predict clinical FK response, MIC data against different fungal isolates can help ophthalmologists determine the most suitable treatment (Kredics et al., 2015). Due to the wide spectrum of fungi causing FK, varying susceptibility patterns are not surprising (Mahmoudi et al., 2018). The MIC results in this study showed that amphotericin B had the lowest MIC_90_ against *Fusarium* species (n=34), followed by voriconazole, posaconazole and natamycin. There were no significant differences in antifungal sensitivity between *Fusarium* species. In our study, we found posaconazole had the lowest MIC_90_ against *Aspergillus* species (n=28), followed by caspofungin, voriconazole, and amphotericin B. Posaconazole had the lowest MIC_90_ when tested on *Candida* species (n=14), subsequently followed by miconazole, voriconazole, flucytosine, fluconazole, amphotericin B and caspofungin. Compared to *Fusarium* species, *Aspergillus* and *Candida* species were inhibited at low MIC values by azoles. As expected, these results support a prior study on *in vitro* susceptibility of fungal isolates (Lalitha et al., 2007). Against *S. apiospermum* (n=4), voriconazole and miconazole had the lowest MIC_90_ followed by posaconazole, caspofungin, natamycin, and amphotericin B. Natamycin had the lowest MIC_90_ among the drugs tested on dematiaceous fungi, followed by posaconazole, caspofungin, voriconazole, amphotericin B, and miconazole. Results of our study are similar to those found by Xie et al. (Xie et al., 2008) and Al-Hatmi et al. (Al-Hatmi et al., 2016) regarding *in vitro* susceptibilities of isolates from keratitis. Contrary to our findings, O’day et al. (O'day et al., 1986) stated that amphotericin B was ineffective against *Fusarium* species (139). According to Hassan et al. (Hassan et al., 2015) amphotericin B, voriconazole, fluconazole, and natamycin proved to be potential antifungal agents for treating human *Fusarium* keratitis. Though susceptibility testing is more popular for bacterial than fungal disease, fungal susceptibility testing is becoming more credible in the literature. According to several studies, infections caused by susceptible isolates respond to therapy better than infections caused by resistant isolates (Lalitha et al., 2007). Therefore, drug susceptibility tests *in vitro* can provide valuable information on the proper treatment of fungal infections, particularly for specific antifungal drugs that interact significantly with fungi.

## Conclusion

It is well-known that corneal damage is a major risk factor in FK. The most at risk group is young and middle-aged farmers due to the increased exposure to corneal damage caused by contaminated crops. Using a diagnostic method that is highly sensitive and specific can enable early initiation of antifungal therapy to allow for a complete recovery within a short period of time. The MIC results indicate that amphotericin B may be appropriate for treating FK caused by *Fusarium* species. Amphotericin B, voriconazole, posaconazole, and caspofungin may be good options in cases of *Aspergillus* keratitis. FK caused by *Candida* spp. can be treated with flucytosine, voriconazole, posaconazole, miconazole, and caspofungin and *S. apiospermum* can be treated with voriconazole and caspofungin. Keratitis caused by the dematiaceous fungi may require the use of antifungals such as amphotericin B, posaconazole, and caspofungin. Research that investigates the relationship between *in vitro* susceptibilities and *in vivo* clinical outcomes will be important for the treatment of fungal keratitis.

## Data availability statement

The raw data supporting the conclusions of this article will be made available by the authors, without undue reservation.

## Ethics statement

This study was approved by the ethics committee of the Tehran University of Medical Sciences (IR.TUMS.MEDICINE.REC.1400.304). The patients/participants provided their written informed consent to participate in this study.

## Author contributions

MS, Az, SK, FA, ZA, AM, MA, and SR collected the data and drafted the manuscript. S.K, MS reviewed the manuscript from the clinical point of view. SK supervised the work and reviewed the manuscript from the microbiological point of view. CO performed susceptibility testing. MT-K performed the molecular identification of fungal strain. RD, SH, FA, and SA reviewed the manuscript from the microbiological point of view. MA and ZA helped with collecting fungal strains and the data. SM and SK reviewed the manuscript from the microbiological point of view and helped with editing the manuscript. SR and MA helped with collecting the data. PV and SK reviewed the manuscript from the microbiological point of view and helped with editing the manuscript. All authors contributed to the article and approved the submitted version.
